# Microbubble Mediated Thrombus Dissolution with Diagnostic Ultrasound for the Treatment of Chronic Venous Thrombi

**DOI:** 10.1371/journal.pone.0051453

**Published:** 2012-12-12

**Authors:** Shelby Kutty, Juefei Wu, James M. Hammel, Feng Xie, Shunji Gao, Lucas K. Drvol, John Lof, Stanley J. Radio, Stacey L. Therrien, David A. Danford, Thomas R. Porter

**Affiliations:** 1 Joint Division of Pediatric Cardiology, University of Nebraska College of Medicine/Creighton University, Children’s Hospital and Medical Center, Omaha, Nebraska, United States of America; 2 Department of Internal Medicine, Section of Cardiology, University of Nebraska Medical Center, Omaha, Nebraska, United States of America; 3 Division of Cardiovascular Surgery, University of Nebraska Medical Center, Omaha, Nebraska, United States of America; 4 Department of Pathology and Microbiology, University of Nebraska Medical Center, Omaha, Nebraska, United States of America; Osaka University Graduate School of Medicine, Japan

## Abstract

**Background:**

Central venous catheter **(**CVC) thrombi result in significant morbidity in children, and currently available treatments are associated with significant risk. We sought to investigate the therapeutic efficacy of microbubble (MB) enhanced sonothrombolysis for aged CVC associated thrombi *in vivo.*

**Methods and Results:**

A model of chronic indwelling CVC in the low superior vena cava with thrombus *in situ* was established after feasibility and safety testing in 7 pigs; and subsequently applied for repeated, sonothrombolytic treatments in 9 pigs (total 24 treatments). Baseline intracardiac echocardiography (ICE, 10.5F, Siemens), fluoroscopy and saline flushing confirmed the absence of any pre-existing CVC thrombus. A thrombus was then allowed to form and age over 24 hours. The created thrombus was localized and measured by ICE, and transthoracic image guided high mechanical index (MI) two-dimensional US treatments (1.1–1.7 MI; iE33, Philips) applied intermittently whenever intravenously infused MBs (3% MRX-801; NuVox) were visualized near the thrombus (n = 10; Group A). Control pigs (n = 10; Group B) received US without MB. All treatments were randomized. Post-treatment thrombus area by ICE planimetry was compared with pre-treatment measurements. Thrombus area measurements before and after treatment were 0.22 and 0.10 cm^2^ respectively in Group A; compared to 0.24 and 0.21 cm^2^ in Group B (p  = 0.0003). Effectiveness of longer duration US and MB thrombolytic treatments were studied (n = 4), which suggested that near complete thrombus dissolution is possible. No pulmonary emboli, alterations in oxygen saturation, or hemodynamics occurred with either treatment.

**Conclusions:**

Guided high MI diagnostic US+systemic MB facilitates reduction of aged CVC associated thrombi *in vivo*. MB enhanced sonothrombolytic therapy may be a non-invasive safe alternative to thrombolytic agents in treating thrombotic CVC occlusions.

## Introduction

Image guided high mechanical index (MI) impulses from a diagnostic ultrasound (US) system during a systemic microbubble (MB) infusion have the potential to dissolve intravascular arterial thrombi without the need for fibrinolytic therapy [1]. Cavitation and resultant shear forces are proposed mechanisms for sonothrombolysis [2].The mechanical effect of cavitation leads to axial fluid acceleration, resulting in acoustic streaming and the creation of high-velocity flow gradients that penetrate and destabilize the structure of the thrombus. This microstreaming phenomenon appears to be one of the mechanisms for sonothrombolysis *in vitro* [2]–[4]. Since contrast MBs serve as nuclei for cavitation, the peak negative pressure threshold required to induce cavitation is lowered when they are present within the field of insonation [2], thereby improving the thrombolytic ability of US.

We recently demonstrated that guided high MI impulses from diagnostic US and MB were successful at dissolving aged venous thrombi within pediatric sized catheters *in vitro*, due to shearing and dissolution of micro fragments from the outer surface of the thrombus [5]. This was the first demonstration of the effectiveness of this technique in older venous thrombi that typically form in chronic indwelling central venous catheters (CVC), or in deep venous thromboses. Because CVC in the clinical setting are often not completely occluded, MB can be infused through the central lumen of these catheters. Therefore, unlike other clinical applications in which only small concentrations of systemically administered MB reach the thrombus because of low flow, MB can be directly infused into the thrombosed CVC enabling a relatively high local MB concentration in the majority of cases. Moreover, delivery of targeted transthoracic US to the region of interest is likely achievable in pediatric subjects due to the generally favorable windows and relative proximity of systemic vasculature to the chest wall. This can potentially contribute to improved outcomes with sonothrombolytic therapy for CVC occlusion.

Our principal hypothesis was that MB enhanced sonothrombolysis is an effective alternative method for treating aged CVC associated thrombi *in vivo*. We sought to investigate the (a) safety and feasibility (Arm 1) and (b) therapeutic effectiveness (Arm 2) of MB enhanced sonothrombolysis for treatment of aged CVC thrombi *in vivo*. Our secondary goal was to determine if inertial cavitation is a necessary component of thrombus dissolution by analyzing the radiofrequency signals returning from guided high MI impulses. A porcine model was chosen based on our previous work with this model, and demonstrated safety of intravenous MB and transcutaneous US in this model.

## Methods

### Microbubble and Ultrasound Technology

The lipid encapsulated MB formulation MRX-801 (NuvOx Pharma, Tucson, AZ) was used. These MB have a diameter of 1.0±0.1 µm, and concentration of 1.5 to 3.0×10^10^/ml. The MB infusion was prepared by diluting 2 ml of the MRX-801 in 100 ml of 0.9% saline and infused at a rate of 1.0 ml/min. The US system (iE33, Philips Medical Systems, Andover, MA) and diagnostic transducer (S5–1, Philips) used are both commercially available. The US system had low MI contrast sensitive imaging pulse sequences (Power Modulation) that were applied in between high MI impulses to assist in the detection of MB. Thereby, the application of brief high MI impulses (1.1–1.7 MI) was guided (5 seconds on, 2 seconds off) to allow replenishment of MB within the pre-specified region of interest. The US system was also equipped with a radiofrequency data acquisition board to serve as an array-based passive cavitation detector. Thereby, recording and analysis of the beam-summed radiofrequency data of signals backscattered by the contrast MB were obtained in studies during the MB infusion and high MI applications.

### Establishment of *in vivo* CVC Model (Arms 1 and 2)

The study was approved by the Institutional Animal Care and Use Committee of the University of Nebraska Medical Center and was in compliance with the standards in the Guide for the Care and Use of Laboratory Animals. The establishment of a chronic animal model of CVC in Arms 1 and 2 was accomplished as below. Each procedure was a survival study and necessitated sterile fields and techniques. The pig was fasted overnight and pre-anesthetized with an intramuscular mixture of Telazol (4.4 mg/kg), Ketamine (2.2 mg/kg), and xylazine (2.2 mg/kg). Intramuscular atropine (0.05 mg/kg) was used to dry oral-tracheal secretions and prevent bradycardia during intubation. Following placement of a venous line in the lateral or medial auricular vein, the pig was intubated and isoflurane inhalation anesthesia (induction at 4%, maintained at 1.0 to 1.8%) administered. The animal was placed on a ventilator at a volume of 11 cc/kg of air and at a rate of 15 breaths/minute.

**Figure 1:**
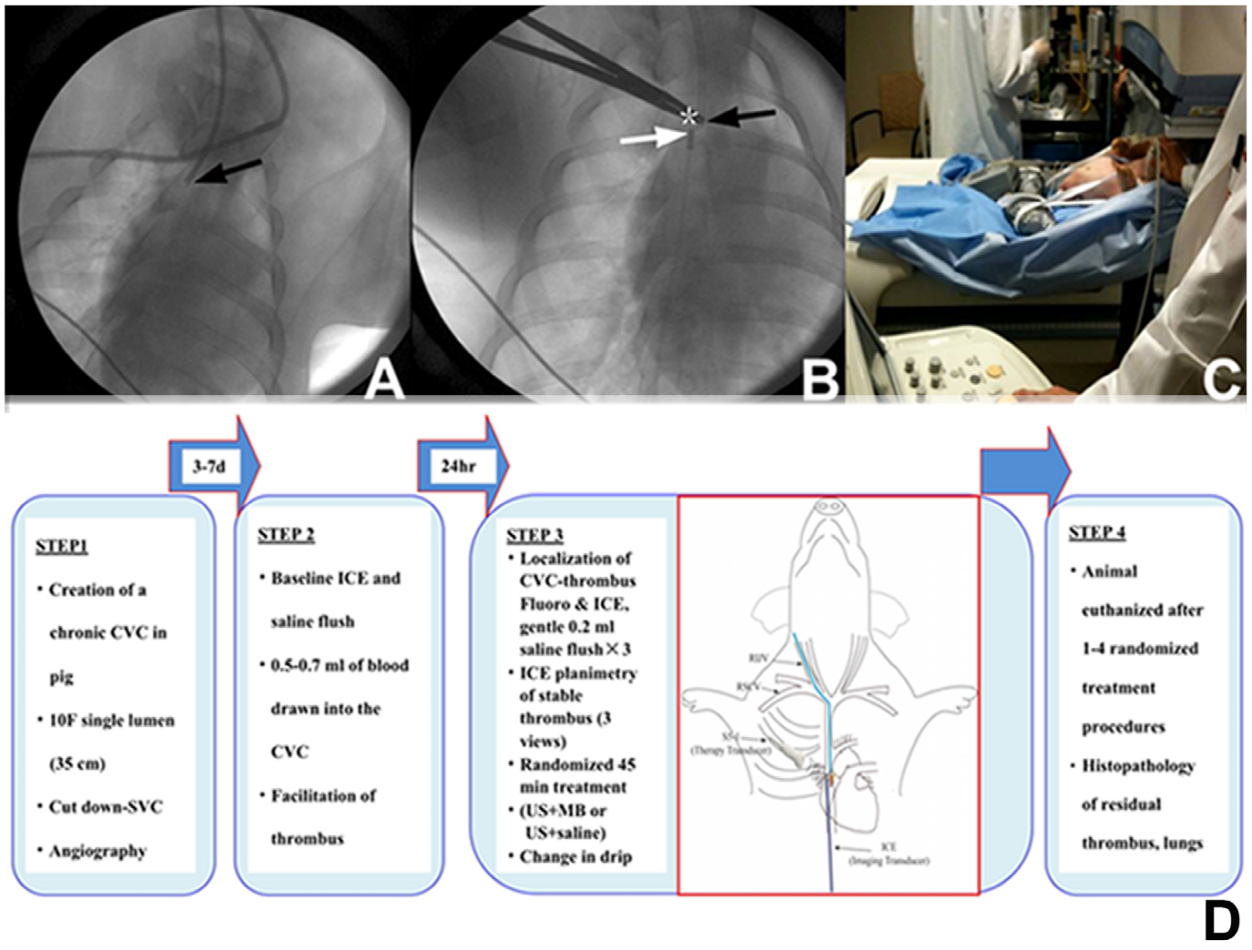
Fluoroscopic images of the central venous catheter and the treatment time line. The central venous catheter is positioned with its tip in the low superior vena cava (Panels A and B; black arrows) and the intracardiac ultrasound transducer is adjacent to the CVC tip (Panel B; white arrow). Asterisk denotes the fluoroscopically localized site of transcutaneous ultrasound application. Panel C shows transcutaneous sonothrombolytic therapy in an animal underneath the SPECT scanner. A schematic representation of the treatment protocol and time line is shown in Panel D.

Indwelling CVC placement was performed via surgical cut-down under full aseptic precautions (JMH). A 10F single lumen CVC (long-term carbothane hemodialysis catheter, 35 cm, Bard Inc., Salt Lake City, UT) was used. After the pig was placed under anesthesia and preparation of surgical site, an incision was made over the jugular furrow of the neck to be continued through the subcutaneous tissue and cutaneous coli muscle. The jugular vein was exposed with blunt dissection, and a short incision made in the skin of the dorsal interscapular region. A trocar was used to tunnel subcutaneously from the ventral incision to the dorsal region of the chest wall and the CVC advanced through the subcutaneous tunnel for placement via surgical cut down in the superior vena cava under fluoroscopic guidance (Cardiovascular mobile digital C-arm system, GE, Milwaukee, WI). The jugular vein was canulated using the modified Seldinger technique, and the CVC positioned with its tip in the low superior vena cava (**Figure 1** **–A and B**). The vessel incision was closed, the exteriorized portion secured to the skin using non-absorbable sutures, and covered by a protective canvas pouch that was sutured to the dorsum of the animal. The animal was recovered from anesthesia with continuous monitoring.

### Facilitation of Thrombus Formation

After an interval of at least 3 days after CVC placement, the animal was placed under anesthesia and an intracardiac echocardiographic (ICE) transducer (10.5–F AcuNav, Sequoia, Siemens Inc., Mountain View, CA) introduced through a femoral venous access and positioned immediately beside the CVC tip. ICE and fluoroscopy were used to image the distal segment and tip of the CVC to confirm the absence of thrombus within the distal segment of the CVC or adherent to the tip, prior to the thrombus induction protocol. After confirmation of no preexisting CVC associated thrombus, the heparin lock solution was withdrawn, and 0.5– 0.7 ml of blood was drawn into the CVC. The animal was allowed to recover, and an aged venous thrombus allowed to form in the distal tip of the CVC over the next 24 hours. Animals then proceeded to either safety testing (Arm 1) or randomized treatments (Arm 2).

### Safety and Feasibility Assessments (Arm 1)

For assessment of safety in the animals that received US-MB treatments, venous blood was drawn (60 ml) for labeling of platelets with In-111 at the time of blood withdrawal for thrombus creation. Approximately 3 hours later, In-111 labeled platelets (200–600 uCi in 5 ml) were administered intravenously to the animal. In-111 labeled platelet scintigraphy of lungs was performed at baseline to examine for pulmonary emboli utilizing a medium energy collimator [Infinia Hawkeye 4 Single photon emission computed tomographic (SPECT) scanner, GE, Milwaukee, WI]. The scan settings were: matrix 128×128, energy peaks at 245 and 171,window 20%, projections anterior-posterior, duration 30 minutes and zoom factor of two. MB enhanced sonothrombolytic treatment was followed by repeat scintigraphy of the lungs using the same imaging parameters as above (**Figure 1C**). Bilateral pulmonary regions on the scintigraphic image were analyzed on Xeleris Functional imaging workstation (GE Healthcare, Milwaukee, WI). The activities expressed as counts per pixel in the lung fields were compared before and after each treatment to assess for pulmonary emboli. Safety was also evaluated by continuously monitoring oxygen saturation, arterial blood pressure, and 3-lead electrocardiograms before, during, and for 30 minutes after US and MB therapy. Activated clotting times were measured before and after each treatment.

### Sonothrombolytic Therapy Protocol (Arm 2)

**Figure 2:**
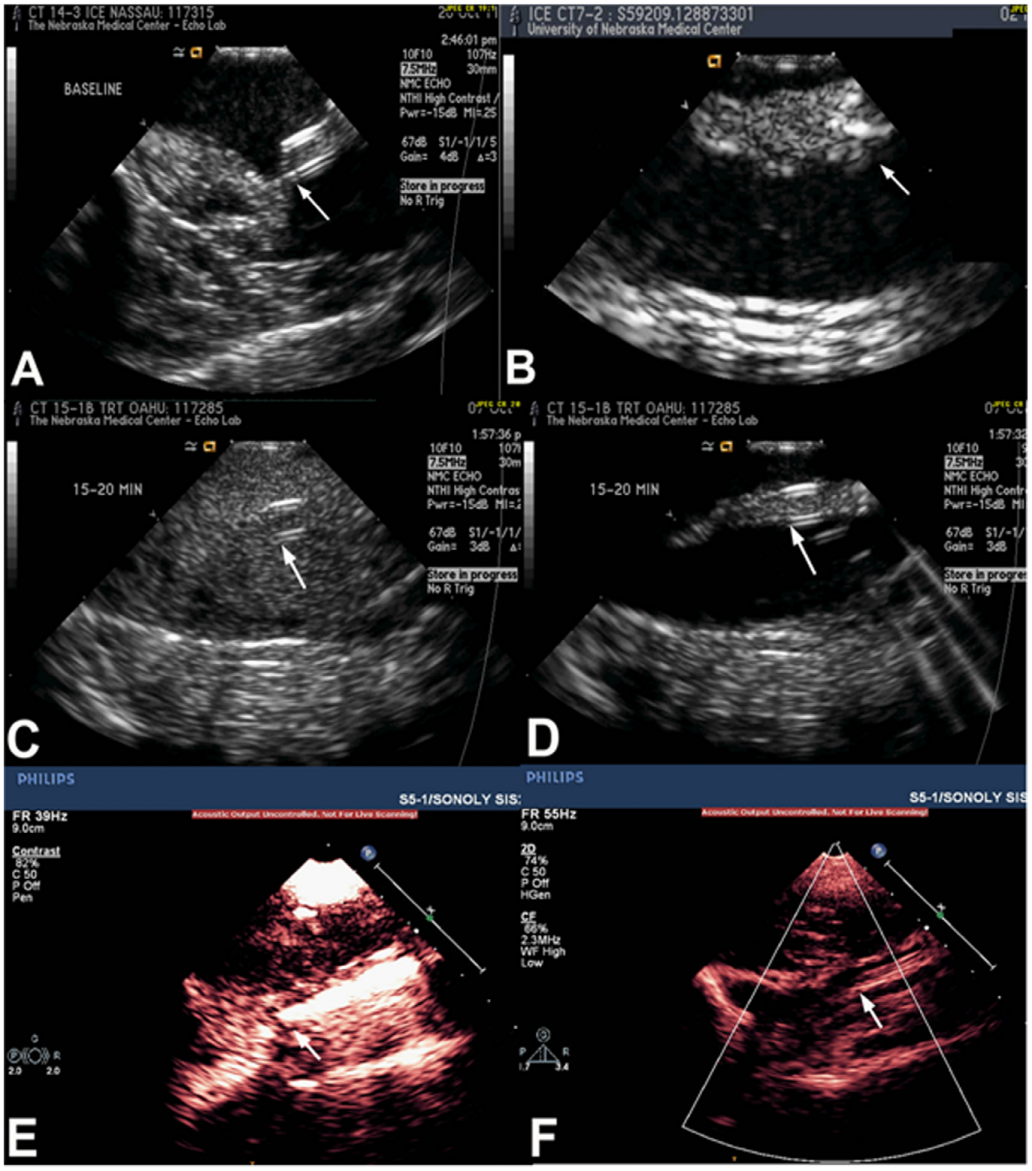
Intracardiac and transcutaneous echocardiographic images during treatment. Panel A demonstrates clean tip of the CVC (arrow) at baseline without any adherent thrombus. Panel B shows a stable thrombus formed at the CVC tip (arrow) 24 hrs after withdrawing 0.5–0.7 milliliters of blood into the CVC. Panels C and E show intracardiac (ICE) images demonstrating high concentration of microbubbles within the distal end and around the CVC in the superior vena cava on low MI imaging. Panels D and F show rapid and complete clearing of microbubbles with the application of high mechanical index pulse sequences to insonify the CVC tip (white arrows).

The time line of the treatment protocol is schematically shown in **Figure 1D**. Twenty-four hours after withdrawal of blood into the CVC, the pig was returned to the laboratory. The CVC tip was localized using fluoroscopy and ICE via the Acunav transducer positioned immediately beside the CVC tip (**Figure 2A**). The CVC was checked for flow and presence of thrombus at it tip, and any thrombus within was gently flushed through with two 0.2 ml saline flushes. Treatments were performed only in the presence of a stable thrombus at the CVC tip that was quantifiable using ICE (**Figure 2B**). Baseline images of the thrombus were then obtained with ICE followed by transcutaneous imaging of the CVC and thrombus using the imaging system (S5–1, iE33, Philips). Randomized treatments were then assigned (US guided high MI impulses during MB infusion or US alone as described below). The oxygen mixture was kept at 24% during each treatment. Venous samples for activated clotting times were obtained before initiation of treatment and at approximately 30 minutes into it. The animal was recovered from anesthesia after each treatment.

The two randomized groups tested were Group A: Intermittent guided sub-clavicular high MI 2-dimensional US (20 second long pulse, frame rate 50–60 ms) pulses applied (5 seconds on, 2 seconds off); or Group B: high MI 2-dimensional US pulses with 0.9% saline infusion instead of MB. MB or saline infusions were delivered through both the CVC and a peripheral intravenous line. The frequency and pulse duration for therapeutic US application was chosen based on previous *in vitro* work from our laboratory. [6] High MI pulse sequences were applied to insonify the CVC tip with its adherent thrombus for 5 seconds, which was then interrupted and the transducer switched to low MI imaging for 2 seconds while MB concentration was visually replenished around the CVC (**Figure 2** **-panels C, D, E and F**). Continuous ICE imaging was performed throughout the treatment period. In the MB treatment group, the ICE was used to ensure that the high MI impulses resulted in MB destruction. All treatments were for 45 minutes. Oxygen saturation, arterial blood pressure, and 3-lead electrocardiograms were continuously monitored before, during, and for 30 minutes after therapy. Planimetry of thrombus area on ICE (average of three measurements) before and after treatment was chosen as the end point to quantify thrombus reduction with time between the two groups. A blinded reviewer (SG) who had no knowledge of treatment assignment and who was unaware of whether images on ICE were before or after treatment, performed all planimetry measurements at a later date. Treatment success was defined as a >50% reduction in planimetered thrombus area on ICE imaging at 45 minutes of treatment.

**Figure 3:**
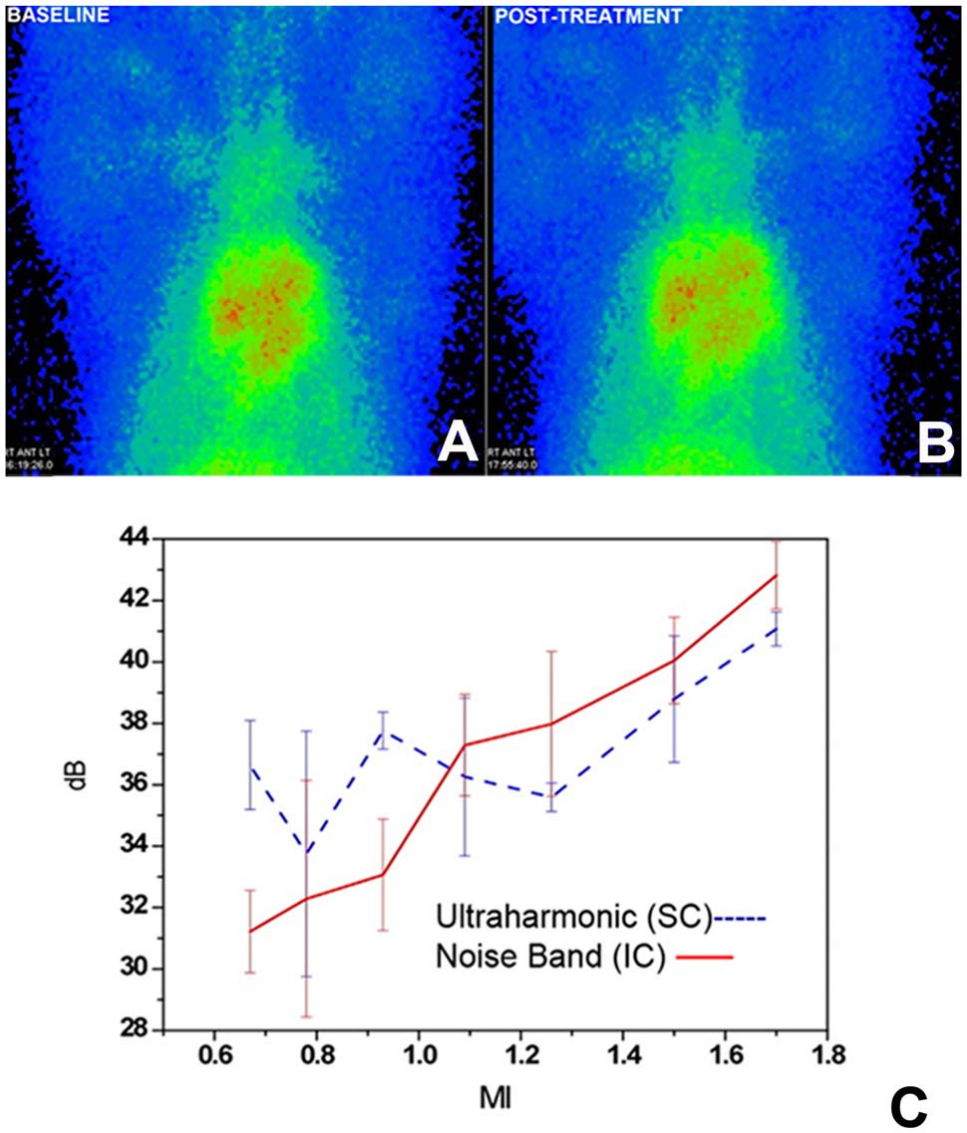
Images of Platelet scintigraphy and Cavitation quantitation. Panels A and B shows bilateral pulmonary regions on In-111 platelet scintigraphy before and after MB and US treatment showing no evidence of emboli. Panel C shows quantitation of the relative contribution of stable vs. inertial cavitation from the treatment area demonstrating the spatially averaged cavitation intensities as a function of mechanical index.

Following the completion of every randomized study, any residual thrombus was removed with tissue plasminogen activator and gentle flushing. The animals were then removed from anesthesia, and extubated. At least one week was allowed before any repeat studies. In between thrombus creation and treatments, the CVC was filled with 20 ml of 0.9% saline followed by 1.0 ml heparin (1,000 U/ml) during weekdays and 5 ml each weekend to maintain patency until the next treatment. Clearance of residual CVC thrombi from previous treatments was confirmed on ICE images performed 24 hours prior to creation of thrombus for the next randomized treatment. The animal was euthanized after 4 treatment cycles or when femoral vein access was no longer accessible. The CVC with the entire segment of the superior vena cava was removed and placed in 10% neutral buffered formalin immediately. Sections of superior vena cava with CVC were processed according to standard tissue processing techniques. Tissues were embedded in paraffin wax, sectioned and stained with hematoxylin and eosin for microscopy. At this point, histopathologic examination of the lungs and CVC were performed to examine for any residual CVC associated thrombus and pulmonary emboli.

**Figure 4:**
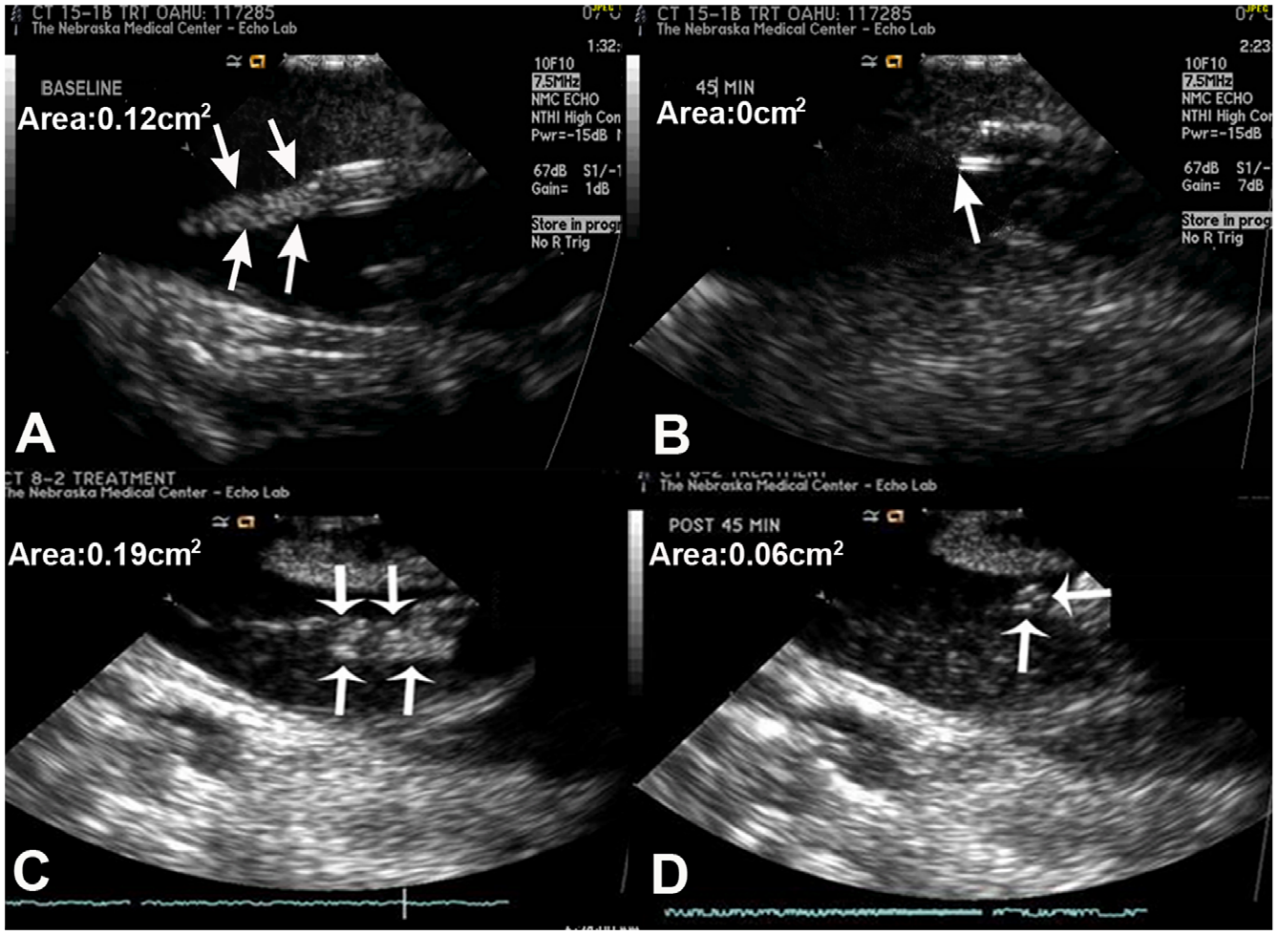
Intracardiac echocardiography before and after treatment. Examples of intracardiac echocardiographic images of the CVC tip before (panels A, C) and after (panels B, D) long pulse ultrasound and microbubble treatments demonstrating varying grades of thrombus reduction after treatment. Thrombus dissolution was complete (Panel B), near complete (Panel D).

### Analysis of Induced Cavitation States and Histology

At the completion of a treatment protocol in 4 animals, returning radiofrequency signals were analyzed during the continuous MB infusion for detection and characterization of cavitation states and their intensity. The aim was to verify which type of cavitation (inertial versus stable) was predominant during the high MI application for dissolution of the CVC thrombi. Custom software performed the spectral analysis of the backscattered data [1], and was used to semi-quantitatively evaluate the relative contribution of stable (as measured by ultraharmonics in the radiofrequency signal) and inertial cavitation (as measured by the noise floor in the radiofrequency spectra) around the region of the CVC tip during each applied high MI impulse.

**Figure 5:**
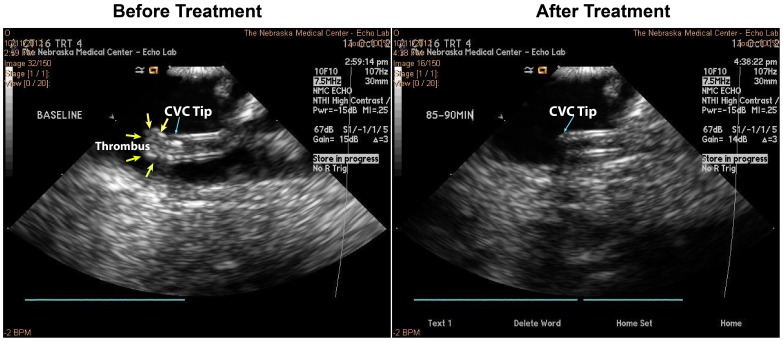
Intracardiac echocardiography from a case of longer treatment duration. Images before and after longer duration (90-minute) ultrasound and micobubble treatment in a case that showed complete thrombus dissolution.

### Applications with Longer Treatment Duration

Four additional experiments were performed to determine if complete thrombus dissolution was possible utilizing longer treatment duration. In these studies, high MI two-dimensional US (20 second long pulse, frame rate 50–60 msec) pulses were applied (5 seconds on, 2 seconds off) during MB infusion for 90 minutes, and comparison of thrombus measurements were made before and after treatment as previously described.

**Figure 6:**
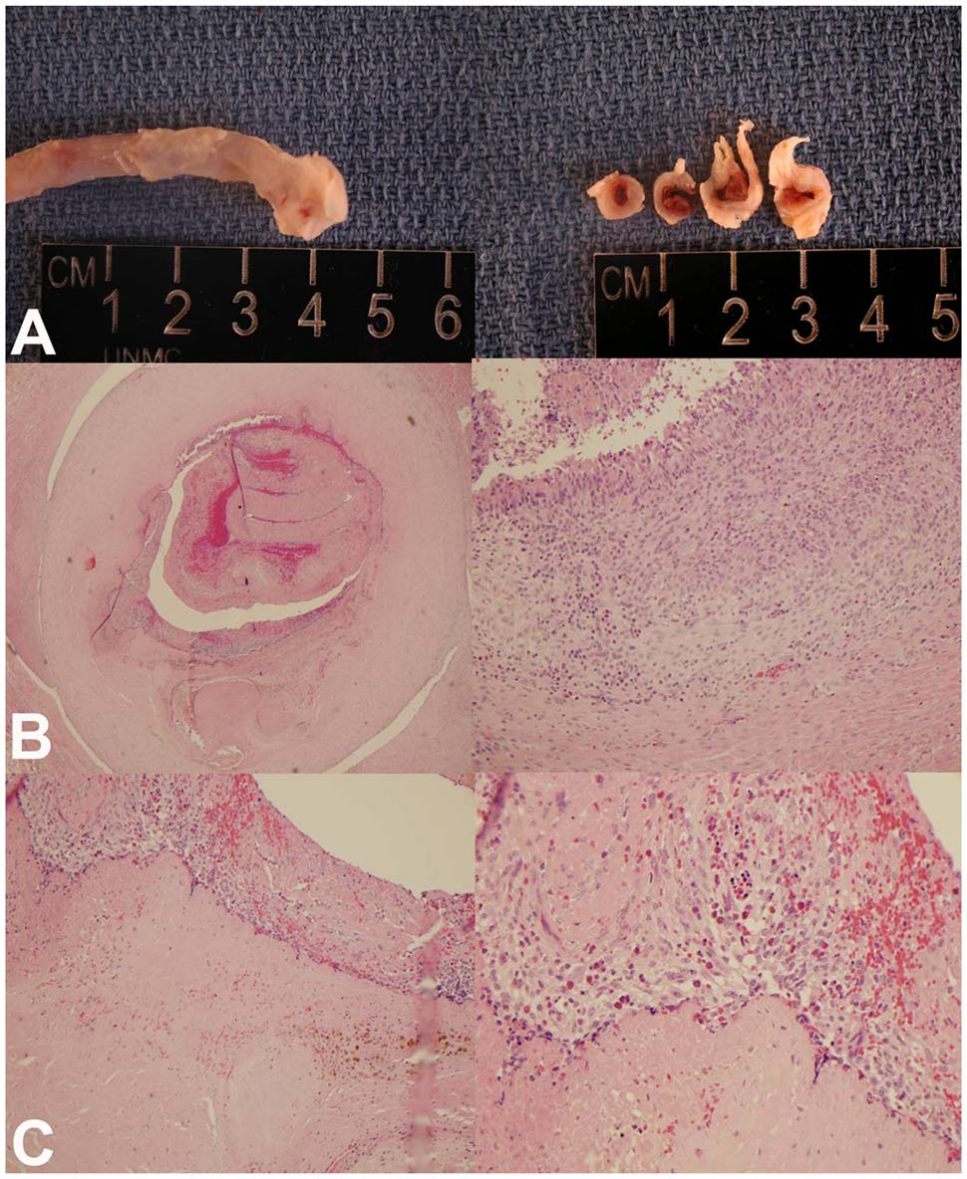
Histologic examination of residual post-treatment thrombus. Panel A shows organized thrombus at the tip of a catheter explanted from the superior vena cava. Panel B shows cross section of the superior vena cava with its proliferated and inflamed intima and organized thrombus within. Panel C demonstrates a combination of newer and older organized thrombi and evidence of chronic inflammatory cells including eosinophils on light microscopy.

### Statistical Analysis

Mean, standard deviation, median and ranges were determined for continuous variables. Paired t test was used for comparison of planimetered thrombus area on ICE before and after randomized treatments. A p value <0.05 was considered significant. Statistical analyses were performed using Minitab 16.0 software (Minitab Inc., State College, PA).

**Figure 7:**
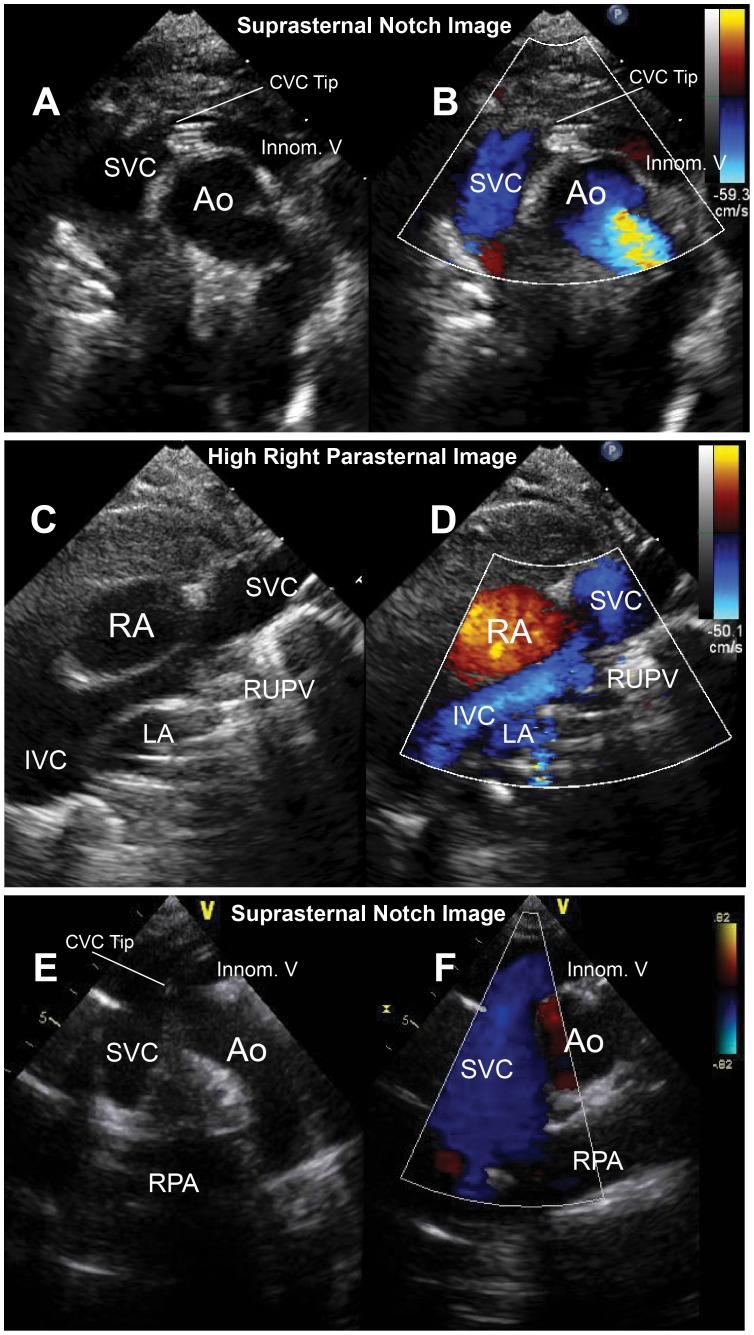
Representative images of superior vena cava and central venous catheter visualization in infants and children. Panels A-D show suprasternal notch and high right parasternal 2-dimensional and color Doppler images of the superior vena cava in an infant. Arrows show the central venous catheter tip. Panels E and F demonstrate the superior vena cava and central venous catheter tip in a 14-year old child.

## Results

### Feasibility and Safety Assessment

Between October 2010 and October 2011, a total of 16 pigs had placement of the CVC (7 for safety and feasibility testing; 9 for treatment protocols). **Figure 3** **–A and B** shows an example of lung scintigraphy before and after treatment using radionuclide scintigraphy (In-111 platelet labeling). There was no evidence of pulmonary emboli detected by this method as a consequence of MB and US treatments in the 7 pigs that underwent these feasibility assessments.

**Table 1:** Summary of hemodynamic data in all animals prior to, during and for 30 minutes following treatment.

Parameters		Group A(2D US*+ MB†)	Group B (2DUS+saline)	*p* value
Before Treatment	HR‡	96±18	93±8	0.44
	SBP§ (mmHg)	98±11	105±20	0.40
	DBP^||^ (mmHg)	50±7	60±17	0.15
	SpO_2_^#^ (%)	100±0	99±0.3	0.34
	ACT**	118±9	114±8	
During Treatment	HR	88±7	88±11	0.98
	SBP (mmHg)	111±14	113±17	0.84
	DBP (mmHg)	67±11	66±12	0.78
	SpO_2_(%)	94±3	96±3	0.19
After Treatment	HR	76±10	85±16	0.13
	SBP	112±17	113±9	0.92
	DBP	66±11	67±7	0.63
	SpO2 (%)	99±3	98±3	0.38
	ACT	114±6	111±11	

* ultrasound,

† microbubble.

‡ heart rate,

§ systolic blood pressure,

||diastolic blood pressure,

#systemic oxygen saturation,

** activated clotting time.

### Randomized Sonothrombolytic Treatments (Arm 2)

Twenty randomized treatments were applied for CVC thrombi in 8 pigs, with each pig being used for a range of one to four randomized treatments. These consisted of 10 MB and US (Group A) treatments and 10 US alone treatments (Group B). Six treatments achieved a greater than 50% reduction in thrombus size in Group A, while this occurred only once in Group B (p = 0.03). The mean ± SD of thrombus areas before and after treatment were 0.22±0.06 and 0.10±0.06 cm^2^ respectively in Group A; compared to 0.24±0.09 and 0.21±0.08 cm^2^ in Group B (p  = 0.0003). The change in area (cm^2^) with treatment was 0.12±0.03 in Group A and 0.03±0.02 in Group B; Group A exhibited a 54±22% reduction in thrombus size compared with 12±8% in Group B (P  = 0.01). **Table 1** summarizes the hemodynamic measurements in all animals just prior to, during and for 30 minutes following treatment. There were no differences in these parameters among the groups with any of the randomized therapies. There were no arrhythmias encountered. The activated clotting times did not change during therapy in either group. **Figure 4** shows 3 examples of ICE images of the CVC tip demonstrating thrombus reduction after treatment. In the 4 experiments performed with longer duration US and MB treatments in 1 pig, the mean thrombus area before and after treatment were 0.23 and 0.07 cm^2^ respectively, indicating a 70% reduction in the thrombus size. Of these 4 treatments, two resulted in near complete resolution of thrombi (**Figure 5**). **Figure 6** shows an example of the histology of the residual thrombus at the tip of a CVC in the superior vena cava. These demonstrated organized recent thrombi with proliferative inflamed intima as well as evidence of chronic inflammation including eosinophils in all animals sacrificed.

### Radiofrequency Analysis of Therapeutic Impulses

**Figure 3C** represents the spatially averaged intensities of the ultraharmonic and noise band spectra of radiofrequency signals from the region of the CVC tip during treatment. It was observed in the four animals tested that predominantly inertial cavitation was induced at the site of the CVC thrombus at the therapeutic MI of 1.1, as indicated by the magnitude of the noise floor on the spectral display.

## Discussion

Central venous and arterial catheters are widely used in the care of pediatric patients and are a major source of thrombo-embolic disease [7]. The presence of an indwelling CVC is the single most important risk factor for childhood venous thrombo-embolism [7]–[9], a growing health problem associated with significant acute mortality and chronic morbidity [7]. The incidence of symptomatic CVC-related thrombosis has been reported to vary between 1.2 and 13.0%. Effective treatment to eliminate the thrombus has benefits beyond simple salvage of CVC functionality because it may prevent complications such as pulmonary emboli and infections. Since CVC-related thrombosis and infections are interrelated, interventions designed to reduce thrombus burden have the potential to also reduce CVC-related infections [10].

Optimal therapy for CVC-associated thrombus or venous thromboembolism remains poorly defined. Current management options in children are limited and imperfect: either remove the CVC or leave the CVC in place and use thrombolytic agents. Removal of the CVC carries special challenges in children because of the limited alternative placement sites in patients who commonly require long-term uninterrupted access. Moreover, insertion of a new CVC may further increase thrombo-embolic risk. [11] Use of thrombolytic drugs in children, however, can be associated with risks of major hemorrhage, reportedly as high as 40% [12]. The safety and efficacy of thrombolytic agents has not been established in children [13]. Recent guidelines therefore discourage the routine use of thrombolytics for treatment of neonatal and pediatric venous thromboembolism, except when major vessel occlusion is causing critical compromise of limbs or organs [14]. The poor risk-benefit profile of the two currently available management strategies testifies to the need for development of a different treatment for CVC related thrombosis in children.

Sonothrombolysis comprises the use of MBs coupled with US to dissolve thrombi without thrombolytic agents, or enhance the effectiveness of exogenous tissue plasminogen activator and the rate of thrombolysis. Diagnostic US and MB treatment regimens have been utilized in animal models to restore myocardial microvascular flow and function in acute myocardial infarction [3], and to recanalize cerebral vessels following ischemic stroke [15]. It has been shown that MB enhances arterial thrombus dissolution *in vivo* in the presence of transcutaneously applied US even in the absence of fibrinolytic agents [16]–[18]. The feasibility of treating deeply located acute intravascular thrombi *in vivo* using intravenous MB and US has also been shown [1], [19]. Recently we created *in vitro* models of aged venous thrombi and demonstrated that successful dissolution is achievable with guided high MI impulses delivered from a diagnostic US transducer, while simultaneously utilizing low MI MB sensitive imaging pulse sequence schemes to detect the MB with the same transducer [5].

In the present study, we describe the application of transcutaneous diagnostic US and intravenous MB treatments to facilitate reduction and resolution of aged CVC-associated thrombi *in vivo*. An image-guided approach was utilized by ensuring the presence of MB around the CVC tip thrombus when the high MI impulses were applied. This therapeutic US and MB application may be particularly advantageous in children because of the proximity of vascular structures to the chest wall with less intervening tissue to limit attenuation of transcutaneously applied US impulses. Because of decreased US attenuation, there is less likelihood of reduction in the peak negative pressure in the region of interest. The superior and inferior vena cavae and the innominate vein are common CVC locations and can be suitably imaged in children from standard suprasternal and subxiphoid pediatric US windows [20], [21]. (**Figure 7**) Thus simultaneous imaging can be performed with the diagnostic transducer, where sensitive low-MI pulse sequence schemes detect the presence of MB around the thrombus and guide the timing of high-MI impulses. The safety and success of these non-invasive therapeutic applications from our porcine model of indwelling CVC increase the potential for translation to the human, and could be a significant new treatment modality. The potential therapeutic implications could extend far beyond the realm of cardiovascular disease, for example into hemato-oncologic disease and renal disease where the use of indwelling catheters are very common.

Importantly, thrombus dissolution in this study was achieved without exogenous fibrinolytic therapy, and without evidence of pulmonary emboli. In the clinical setting, significant thrombus reduction would allow for continued CVC patency and use, while facilitating ongoing thrombolytic treatment. Results from our 90-minute applications suggest that longer treatments would promote further lysis, and could achieve the goal of complete thrombus dissolution. Further studies, possibly incorporating techniques such as ligand attachment to MB (e.g. targeting to fibrin or platelets) that increase their affinity to the surface of thrombi and enhance cavitation effects, may have the potential for improved thrombus reduction during treatment. Further work is also needed to assess the success of this therapy for older CVC thrombi (aged several weeks) and to assess the concomitant effect of low dose tissue plasminogen activator with therapeutic US and MB applications.

### Study Limitations

Parameters of optimal US application for MB enhanced sonothrombolysis, i.e. with maximum therapeutic efficacy and no adverse bioeffects, are unknown. Although there is potential for unwanted mechanical bioeffects at MI>1.0, the effects are likely to be minimal because the US application used in the study was with a commercially available diagnostic transducer, and the high-MI therapeutic pulses were significantly lower than the Food and Drug Administration recommended MI limit (1.9) for clinical use. However, we do recognize that bioeffects can still occur with a transthoracic MI>1.0. The mechanistic data suggested that inertial cavitation was dominant during the high-MI US application for sonthrombolysis of the 24-hour old CVC thrombi. However, the extent of treatment success with stable cavitation, and relative contribution of stable and inertial cavitation for achieving successful MB enhanced sonothrombolysis, was not quantified. A potential disadvantage to the application of sonothrombolysis in the clinical setting is the variable age of CVC thrombi. The histology samples we obtained demonstrated organized thrombi, but with further aging there may be more fibrin cross-linking and clot retraction that may render the thrombus resistant to cavitation dissolution. Finally, although we were able to substantially reduce the thrombus burden at the CVC tip, there was still significant residual thrombus noted in some of the pigs. This may indicate the need for more prolonged applications of US and MB in these settings, which was not tested in the present study.

### Conclusions

Guided transcutaneous high MI diagnostic US during a systemic MB infusion facilitates reduction and resolution of aged CVC associated thrombi *in vivo*. This technique may be a non-invasive safe alternative to thrombolytic agents in treating thrombotic CVC occlusions. Inertial cavitation was dominant during this therapeutic US application. Although ultrasound contrast agents have not been approved for pediatric use, recent clinical trials have indicated they are safe and effective for improving left and right ventricular opacification and endocardial border delineation [22]. The particular contrast agent used for this study was similar to the commercially available agent Definity (Lantheus Medical). Therefore, the success with this non-invasive targeted MB and US therapy in this large animal model indicates it could be safely examined for the management of CVC associated thrombi in patients.
